# Disseminating Truth and Doing Good a La Mode

**Published:** 1885-10

**Authors:** 


### DISSEMINATING TRUTH AND DOING GOOD "A LA MODE."

The manner of doing good, and of disseminating truth, varies according to the notion of the disseminator, and the kind of truth to be disseminated, as well as with the kind of good to be done, and the class or individuals who are to be the recipient. Thus, Ecclesiastics adopt the preaching of the Gospel and the distribution of Bibles as a method of doing good, from their standpoint. The modern philanthropist, with an eye to the more substantial nature of the good, endows a college or founds a hospital. An idea of doing good, once greatly in vogue, is admirably portrayed by Dickens, in the character of Mrs. Jellaby (Bleak House), whose whole soul was in sending red flannel shirts and Bibles to Borrioboola-gah, to the young savages, while her own young savages were crying for bread and falling over the bannisters. An individual philanthropist, a gambler, perhaps, may have an erroneous impression that he is doing good when he slips a five-dollar bill into the hand of a shivering, ill-clad female mendicant at the street corner, or sends a barrel of flour "all unbeknown'st," or a load of coal to Dick Dooley's widow, left forlorn, with six little savages, by the falling of the walls of a burning building on Dick, while he, poor fool, was giving expression to *his* crude idea of good by trying to rescue a child from the fourth story. There are, we say, many ways of doing good, and of disseminating truth; but commend us to the modern, of which we have an illustrated edition just to hand, in the shape of a pamphlet, a reprint with a *photograph*, and a hospital and a college on it.

In this age of progress and civilization there are those who think that if they can do good to an individual, a class, or a community, and at the same time *benefit themselves*, they are doing a double good, and that Heaven's recording clerk should score two points to their credit. This was the idea, no doubt, of the Pharisee, who thought he should *have credit* for giving alms. Why not? If one can do good to one's neighbor and to one's self at the same time, *why not?*

They say great minds run in the same channel. Undoubtedly Professor Windy Bowel, M. D., thought as we do, when he attached his photograph to a reprint of a learned article he had written (full of truths, no doubt), and with another picture of his hospital, and the following extraordinary address to "Dear Doctor," sent them to the medical profession. The diagnosis of this hermaphrodite pamphlet would have been obscure but for the address :

Dear Doctor :—It is manifestly the duty of every upright citizen to disseminate truth and to do good. Upon this principle, and in strict accord with *the* ethics, I send you this pamphlet. I do not assume to be wise above others, but nevertheless I have a hospital, any number of boarding houses at my command, and all the latest and most approved facilities for every phase of gynecological practice,[which you fellows haven't got;] therefore you must send such cases as fall into your hands on to *me!* * * * My photograph is designed as a response to the request of ever so many persons whom I shall never meet in this life, and who have expressed a desire to see my face." [! ! !]

Mind you, this pamphlet was not sent to the Heathen, whose soul is pictured as starving for "the truth," and on whose dark mind the light of gynecology never shone ; nor yet to the suffering female, with lacerated perineii or cervices, whose sad souls are also athirst, and crying out in anguish for gynecological light; Oh, no; *they* could not reciprocate; that would be only a single good, and the recording clerk couldn't see his way clear to a double score for the Professor; but they are sent to the doctors,—to those who have not the "approved facilities" and a hospital; and who are famishing for one brief, soul-snatching glimpse of that benevolent phiz.

We confess we *were* sad to think *we* might never meet him in this life; but now, having been permitted to drink (second hand) at the fountain of truth and goodness, so to speak, to gaze on the broad expanse of that cheek, to metaphorically kiss the hem of the great man's garment, we have arisen, like Sally in the song, and wiped our eye out with our frock, and are happy. We feel better. Our friends all feel better, too, notwithstanding our recent attack of dengue, since they, like ourselves, have seen that ethical pamphlet, pregnant to bursting with truths, and only held down by *the* photo in front, and *the* hospital in rear, like soldiers, fore and aft, guarding a convoy of precious treasure.

Ah, but our souls go out in sympathy with the unfortunate ones who may never hope to meet this modern Father Grimes, that good old man, who, although he has "no malice in his heart, no ruffle in his shirt," has better, far, than these;—he has the "most approved appliances," and a hospital, and scores *and* scores of boarding houses; nor yet to behold even the shadow of that royal countenance! May we not hope that within that distant Aiden, (too commonly called the sweet subsequently) they may clasp this rare and r-idiculous personage, who so taketh the name of ethics in vain?

The medical profession should get up a testimonial for him, even if it be but an humble leather medal.

